# M. Nelaton

**Published:** 1864-11

**Authors:** 


M. Nelaton.—This eminent surgeon has recently received a very handsome gold medal from the Italians resident in Peru, as a token of their gratitude for his attendance on Garibaldi.

